# Endothelial nitric oxide synthase overexpressing human early outgrowth cells inhibit coronary artery smooth muscle cell migration through paracrine functions

**DOI:** 10.1038/s41598-017-18848-z

**Published:** 2018-01-17

**Authors:** Sergio Guber, Talin Ebrahimian, Maryam Heidari, Nicoletta Eliopoulos, Stephanie Lehoux

**Affiliations:** Lady Davis Institute for Medical Research, McGill University, Montreal, Quebec, Canada

## Abstract

Cells mobilized from the bone marrow can contribute to endothelial regeneration and repair. Nevertheless, cardiovascular diseases are associated with diminished numbers and function of these cells, attenuating their healing potential. Gene transfer of endothelial nitric oxide synthase (eNOS) can restore the activity of circulating cells. Furthermore, estrogen accelerates the reendothelialization capacity of early outgrowth cells (EOCs). We hypothesized that overexpressing eNOS alone or in combination with estrogen stimulation in EOCs would potentiate the beneficial effects of these cells in regulating smooth muscle cell (SMC) function. Native human EOCs did not have any effect on human coronary artery SMC (hCASMC) proliferation or migration. Transfecting EOCs with a human eNOS plasmid and/or stimulating with 17β-estradiol (E_2_) increased NO production 3-fold and enhanced EOC survival. Moreover, in co-culture studies, eNOS overexpressing or E2-stimulated EOCs reduced hCASMC migration (by 23% and 56% respectively), vs. control EOCs. These effects do not implicate ERK1/2 or focal adhesion kinases. Nevertheless, NOS-EOCs had no effect on hCASMC proliferation. These results suggest that overexpressing or activating eNOS in EOCs increases their survival and enhances their capacity to regulate SMC migration through paracrine effects. These data elucidate how eNOS overexpression or activation in EOCs can prevent vascular remodeling.

## Introduction

Endothelial damage and dysfunction contribute to the development of atherosclerosis, coronary heart disease and restenosis after angioplasty. Endothelial cells (ECs) usually remain quiescent as a result of contact inhibition. However, with age or after mechanical injury, cells may detach and die or undergo apoptosis, facilitating atherosclerotic lesion formation^1^. Loss of EC function also promotes changes in vascular smooth muscle cell (SMC) proliferation, migration and extracellular matrix production^2^. This issue is a prominent feature of restenosis after angioplasty. Although stents are commonly coated with anti-proliferative agents to hamper the excessive proliferation and migration of smooth muscle cells, these drugs also slow vascular reendothelialization, in part by promoting early outgrowth cell (EOC) apoptosis^3^.

EOCs are premature circulating cells that are derived from the bone marrow and are involved in postnatal vasculogenesis and reendothelialization after endothelial damage^4–6^. EOCs are capable of exerting paracrine functions and differentiating into functional endothelial cells. We and others have shown that EOCs can be recruited to sites of endothelial injury and participate in reendothelialization^7–9^. The therapeutic significance of EOCs is limited because of their low numbers in circulation, particularly in patients with elevated cardiovascular disease risk^10^. Nevertheless, the number and function of circulating EOCs may be influenced favorably by factors such as estrogen levels and statin therapy. Estrogen treatment causes EOCs to secrete more growth factors and facilitates their integration at sites of angiogenesis^11^. Both estrogen and statins have also been shown to act through the eNOS/Akt pathway^12,13^. In the case of estrogen, these effects may involve both genomic processes (increased eNOS transcription) and non-genomic processes (enhanced enzymatic activity of eNOS)^12,14–16^. Appropriately, the reendothelialization and neovascularization capacity of EOCs is increased when eNOS is upregulated^17–19^. Moreover, it was shown that *in vivo* transplantation of EOCs overexpressing eNOS in rats repairs injured vessels by inhibiting neointimal hyperplasia and restoring vascular function^7^. Importantly, two recent clinical trials reported the safety and potential efficacy of endothelial progenitor cells overexpressing eNOS and autologous CD34 positive cell treatments in patients with pulmonary arterial hypertension and left ventricular dysfunction post-STEMI, respectively^20,21^.

Surprisingly, although the beneficial effects of eNOS overexpressing EOCs has been well documented *in vivo*, there is little known on how these cells influence SMCs. We hypothesized that EOCs inhibit SMC proliferation and migration, and that eNOS overexpression would enhance EOC function. In our study, eNOS overexpression was performed either by transfecting EOCs with an eNOS plasmid or by stimulating EOCs with estrogen. We observed that eNOS-overexpressing or estrogen-stimulated EOCs have an increased survival and inhibit migration of SMCs through paracrine effects.

## Results

### EOCs do not affect human coronary artery SMC (hCASMC) proliferation or migration

**Figure 1:**
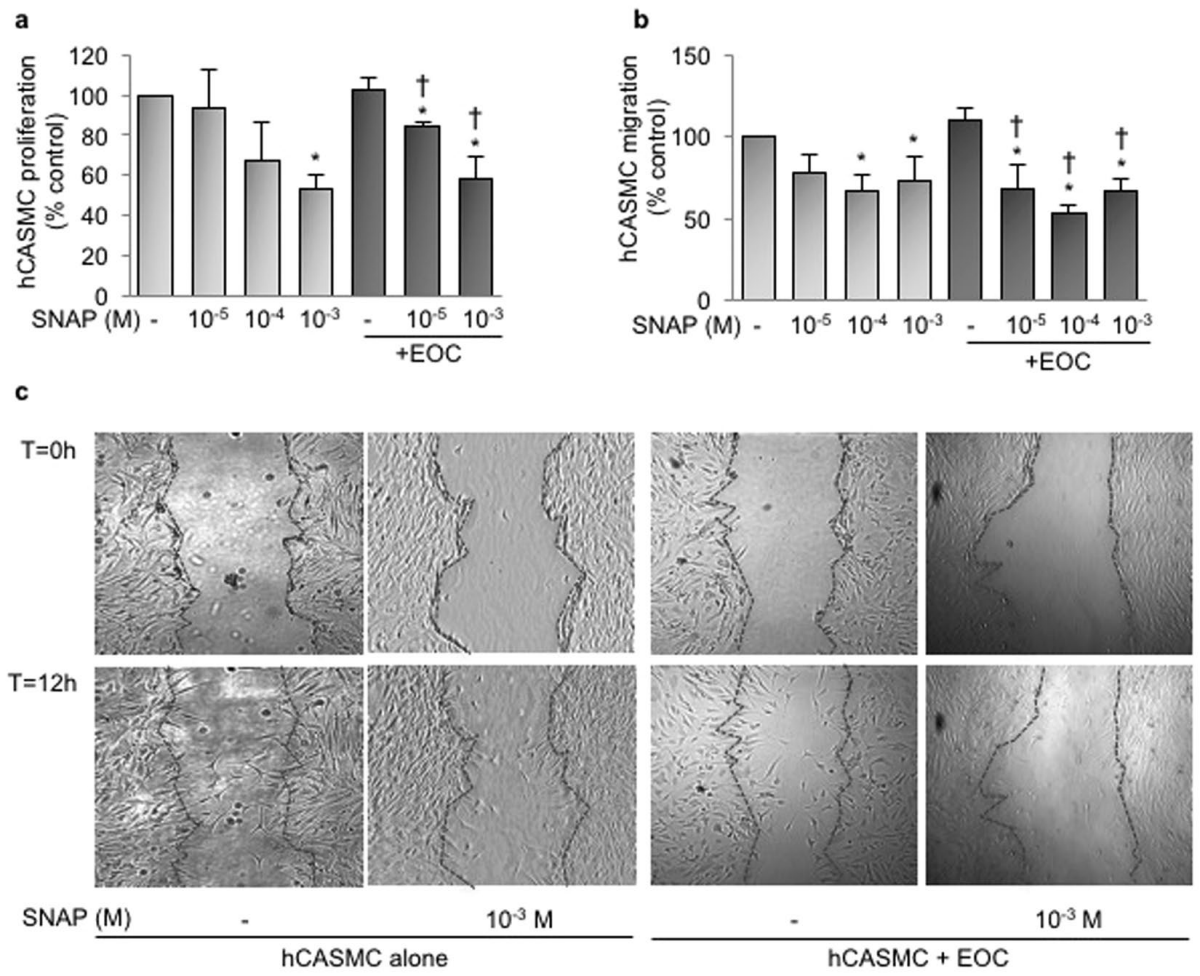
Intact EOCs do not affect SMC proliferation or migration. Human coronary artery smooth muscle cells (hCASMCs), co-cultured or not with early outgrowth cells (EOCs), were stimulated with S-nitroso-N-acetylpenicillamine (SNAP) at different concentrations as indicated. (**a**) hCASMC proliferation was assessed by 24 h BrdU incorporation. (**b**) hCASMC migration was assessed by scratch assay after 12 h. Representative images are shown in (**c**). Data are mean ± SEM of n = 6, expressed as % control (hCASMCs cultured alone, in absence of SNAP treatment (−)). *p < 0.05 vs untreated (−), † < 0.05 vs untreated + EOCs.

Nitric oxide has been shown to decrease proliferation in cultured rat SMCs^22^. To determine if EOCs have a similar effect, human coronary artery SMCs (hCASMCs) were treated with the NO donor S-nitroso-N-acetylpenicillamine (SNAP, 10^−5^–10^−3^ M) and/or co-cultured with 10^6^ EOCs. EOCs were seeded in cell culture well inserts, such that any effects on SMCs would be through paracrine stimulation rather than direct contact. Proliferation was assessed by 24 h BrdU incorporation in hCASMCs. A scratch assay was performed to evaluate hCASMC migration after 8 h. EOCs alone did not have any effect on SMC proliferation or migration (Fig. 1a,b). On the contrary, SNAP decreased the proliferation and migration of SMCs in a dose dependent manner. Nevertheless, hCASMC sensitivity to SNAP was accrued when SNAP was administered in the presence of EOCs.

### Transfection of EOCs with eNOS plasmid increases NO production and cell survival

**Figure 2:**
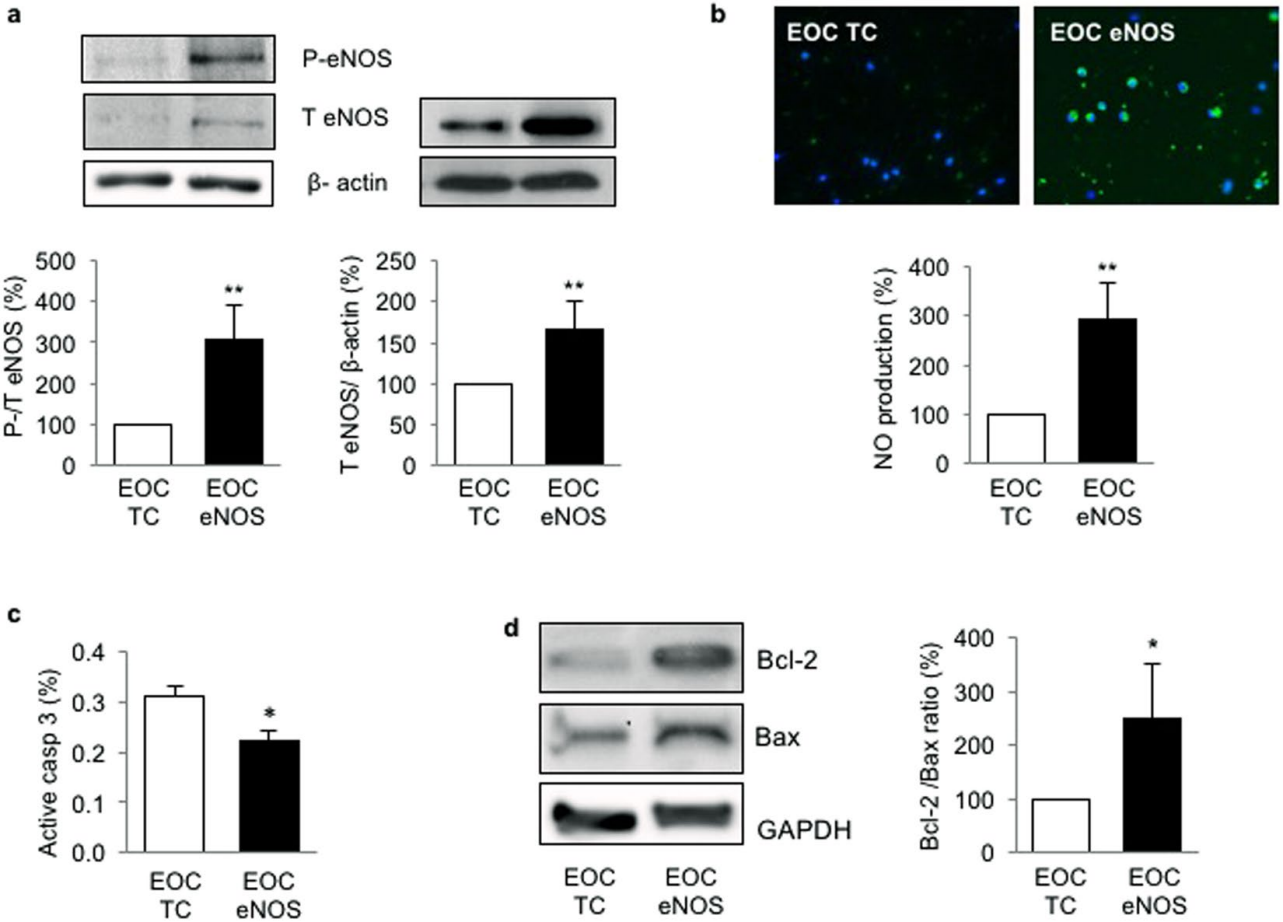
Transfection of EOCs with eNOS plasmid increases NO production and cell survival. Human EOCs were transfected with a transfection control plasmid (EOC TC) or eNOS plasmid (EOC eNOS) for 48 hours. (**a)** Total (T) and phosphorylated (pS1177) (P) eNOS expression was evaluated by western blot. (**b**) Nitric oxide (NO) production was assessed by DAF-FM diacetate, an NO fluorescent probe (green). (**c**) Apoptosis was measured by active caspase 3 flow cytometry. (**d**) The Bcl-2/Bax protein ratio was assessed by western blot and expressed as % of EOC TC. Data are mean ± SEM of n = 6, *p < 0.05 and **p < 0.01 vs EOC TC.

In order to improve the functionality of EOCs, we chose to up-regulate NO synthesis in the cells. Human EOCs were transfected with either eNOS or control plasmid for 48 hours. Both total expression levels of eNOS and eNOS phosphorylation at serine 1177 were increased 3-fold the eNOS-transfected EOCs compared with control transfected cells (Fig. 2a). Accordingly, we also observed a significant increase of NO production in cells transfected with eNOS plasmid (Fig. 2b). Moreover, because NO is a protective factor, we assessed EOC apoptosis and survival. We found a significant decrease in active caspase 3 expression, which is a pro-apoptotic factor (Fig. 2c), and an increase in the Bcl-2/ Bax expression ratio, a molecular index of cell survival, in eNOS-transfected EOCs compared with control transfected cells (Fig. 2d). Analysis of annexin V/propidium iodide staining by flow cytometry further revealed a 17 ± 7% reduction in cell apoptosis among EOC eNOS cells compared with EOC TC. These results suggest that eNOS plasmid transfection of EOCs not only upregulates NO production but also increases their survival.

### eNOS-transfected EOCs decrease migration but not proliferation of hCASMCs

**Figure 3:**
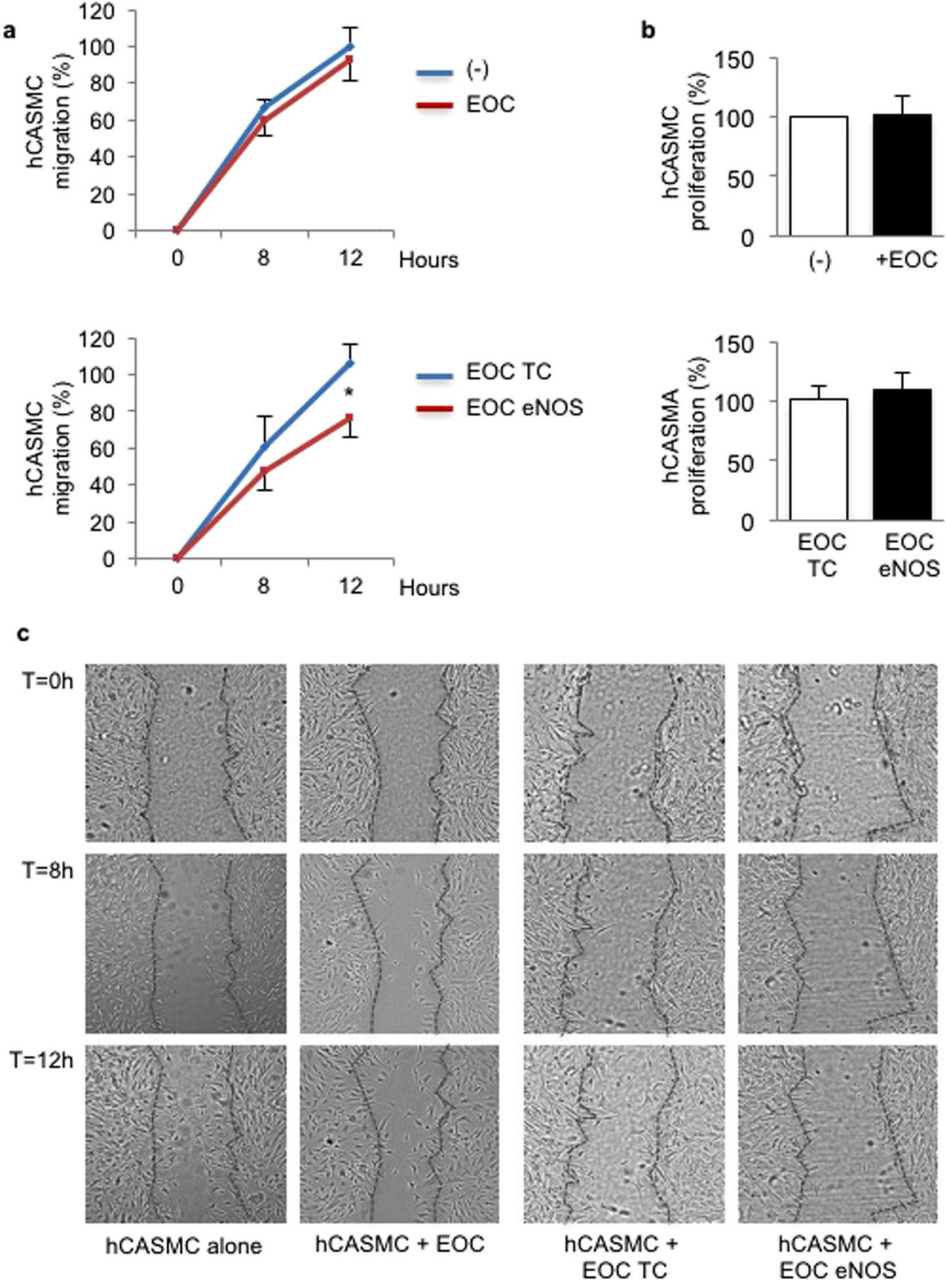
eNOS–transfected EOCs decrease migration but not proliferation of hCASMCs. Human coronary artery smooth muscle cells (hCASMC) were either cultured in the absence of EOCs (−) or co-cultured with human EOCs transfected with a transfection control plasmid (EOC TC) or eNOS plasmid (EOC eNOS) for up to 12 hours (**a**) or 48 hours (**b**). (**a**) hCASMC migration was assessed by scratch assay. Data are expressed as % maximal migration observed in hCASMC cultured in the absence of EOCs. Representative images are shown in (**c**). (**b**) hCASMC proliferation was measured by Ki67 immunostaining as a ratio of total cell number. Data are expressed as % maximal proliferation observed in hCASMC cultured in the absence of EOCs. Data are mean ± SEM of n = 6–7, *p < 0.05 vs EOC TC.

To test whether eNOS overexpressing EOCs regulate hCASMC migration and proliferation, hCASMCs were co-cultured with EOCs transfected with eNOS or control plasmid. As seen in Fig. 3a (upper panel) and in confirmation of Fig. 1, there was no significant difference in hCASMC migration whether alone or in the presence of native EOCs. However, hCASMCs co-cultured with eNOS-overexpressing EOCs migrated less than hCASMCs co-cultured with transfection control EOCs (Fig. 3a, lower panel). These results suggest that NO released by eNOS-transfected EOCs reduces hCASMC migration through paracrine effects. In contrast, we did not observe any effect of eNOS-overexpressing EOCs on hCASMC proliferation (Fig. 3b).

### Estrogen increases eNOS phosphorylationN, NO production and survival of EOCs

**Figure 4:**
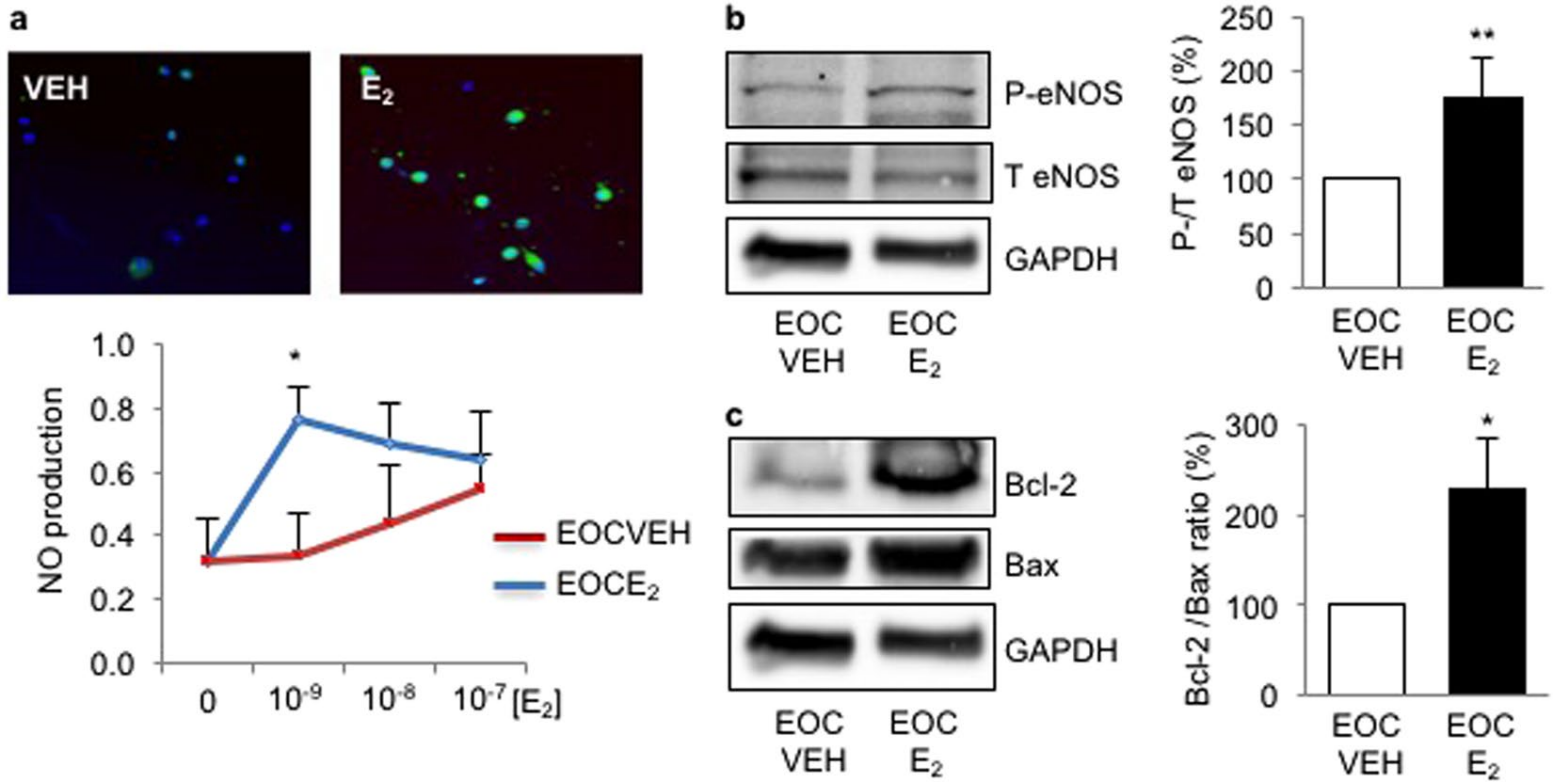
Estrogen increases eNOS phosphorylation, NO production and survival of EOCs. Human EOCs were stimulated with vehicle (VEH) or estrogen (E_2_) at different concentrations (**a**) or at 10^−9^M (**b**,**c**). (**a**) Nitric oxide (NO) production by EOCs was measured by DAF-FM diacetate, a NO fluorescent probe (green). Results are expressed as DAF-FM/Dapi positive cell ratios reported to untreated cells. Representative images of EOCs stimulated with VEH or E_2_ (10^−9^ M) (right). (**b**) Total (T) and phosphorylated (pS1177) (P) eNOS expression was evaluated by western blot. (**c**) Cell survival was assessed by Bcl-2 and Bax protein expression ratio by western blot. Data are mean ± SEM of n = 6, *p < 0.05 and **p < 0.01 vs VEH.

Since NO production has been shown to be regulated by estrogen stimulation, we used estrogen as an alternate approach to up-regulate NO production by EOCs. We started with a dose response study to determine the optimal estrogen (E_2_) concentration to be used, and found that 10^−9^ M E_2_ induced the greatest NO production by EOCs (Fig. 4a). This concentration was used for all subsequent experiments. We observed a significant increase in eNOS phosphorylation (Fig. 4b) as well as Bcl-2/ Bax expression ratio (Fig. 4c) in EOCs stimulated with E_2_ compared with dimethysulfoxide vehicle (DMSO 1:4000). This suggests that E_2_ increases NO production by upregulating eNOS, leading to an increase of EOC survival.

### Estrogen pre-stimulated EOCs decrease migration but not proliferation of hCASMCs

**Figure 5:**
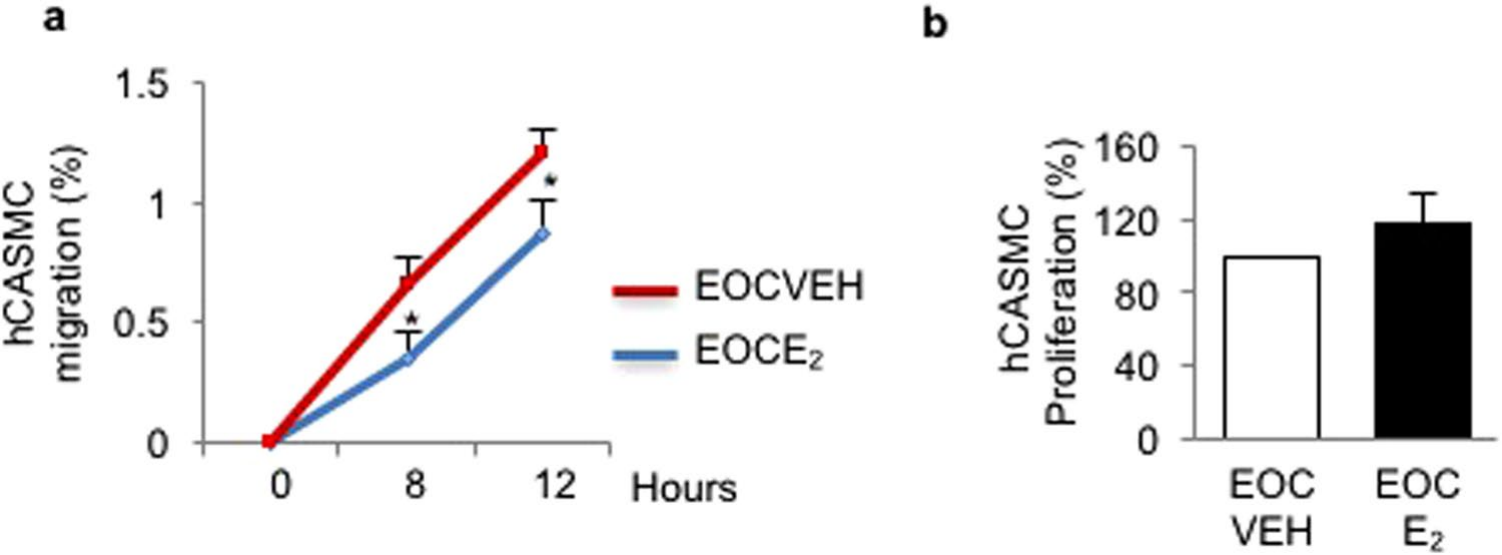
Estrogen pre-stimulated EOCs decrease migration but not proliferation of SMCs. Human EOCs were pre-stimulated with vehicle (VEH) or estrogen (E_2_, 10^−9^ M) for 24 h and co-cultured with human coronary artery smooth muscle cells for up to 12 hours (**a**) or 24 hours (**b**). (**a**) SMC migration was assessed by scratch assay. (**b**) SMC proliferation was measured by Ki67 immunostaining. Data are mean ± SEM of n = 6, *p < 0.05 vs VEH (at same time point).

We showed that eNOS overexpressing EOCs regulate hCASMC migration (Fig. 3a). To determine whether E_2_-stimulated EOCs could have a similar effect, EOCs were pre-stimulated with E_2_, rinsed, and incubated with the E_2_ receptor inhibitor fulvestrant (10^−9^M) prior to co-culture with hCASMCs. We found a significant decrease of hCASMC migration in the presence of E_2_-stimulated EOCs compared with vehicle (Fig. 5a). However, we did not observe an effect on hCASMC proliferation (Fig. 5b). These results suggest that hCASMC migration could be regulated by EOCs pre-treated with E_2_.

### eNOS transfection and E_2_ stimulation have an additive effect on NO production and cell apoptosis in EOCs

**Figure 6:**
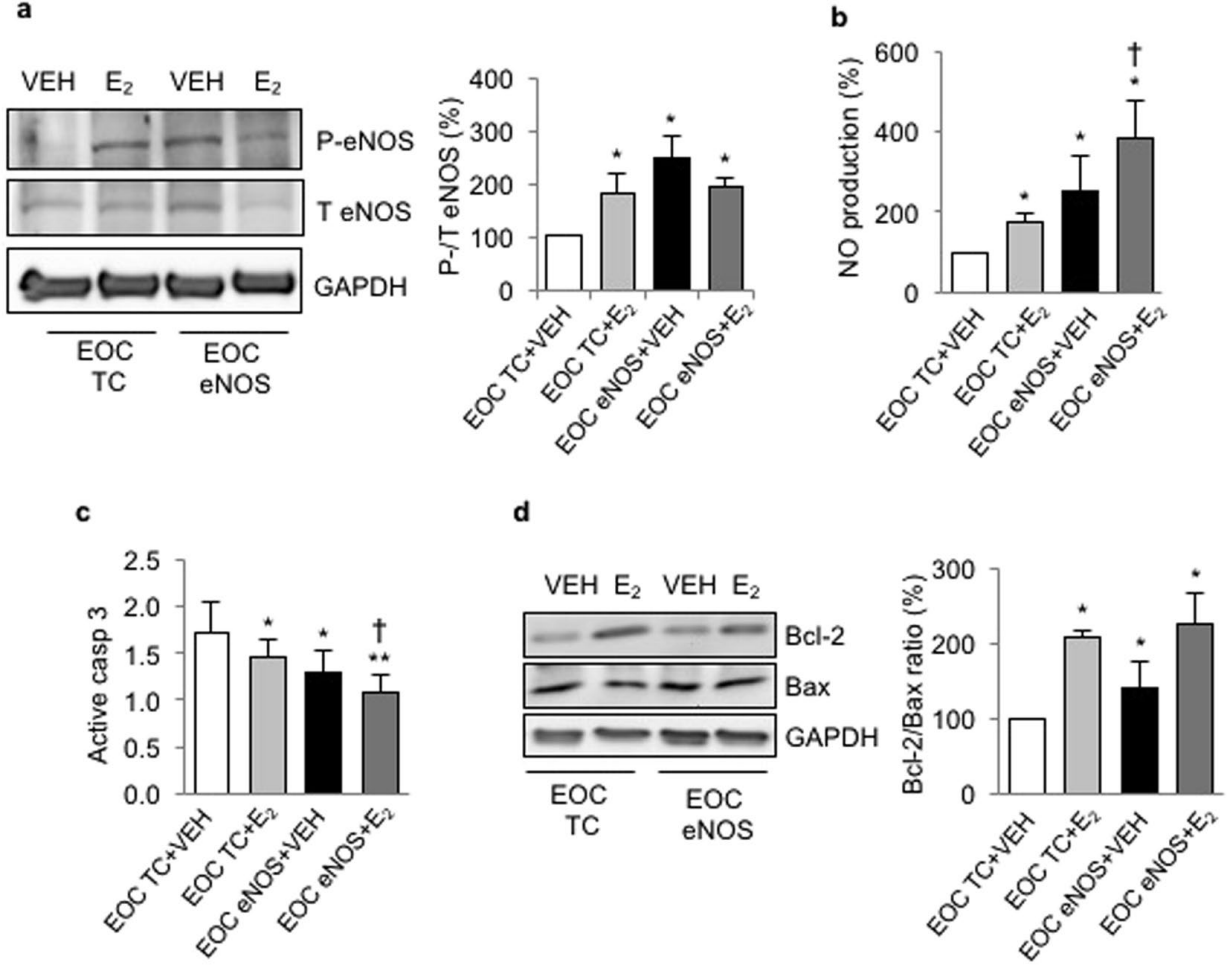
eNOS upregulation by plasmid transfection and estrogen have an additive effect on NO production and apoptosis reduction in EOCs. Human EOCs were transfected with a transfection control plasmid (EOC TC) or eNOS plasmid (EOC eNOS) and stimulated with vehicle (VEH, DMSO) or estrogen (E_2_, 10^−9^M) for 24 hours. (**a**) Total (T) and phosphorylated (P) eNOS (S1177) expression was evaluated by western blot. (**b**) Nitric oxide (NO) production was assessed by DAF-FM, a NO fluorescent probe. (**c**) Apoptosis was measured by active caspase 3 flow cytometry. (**d**) Cell survival was assessed by Bcl-2 and Bax protein expression ratio by western blot. Data are mean ± SEM of n = 6, *p < 0.05 and **p < 0.01 vs EOC TC + VEH, †p < 0.05 vs EOC TC + E_2_.

To determine whether there is an additive effect of eNOS transfection and E_2_ stimulation in EOCs, cells were transfected with eNOS plasmid and stimulated with E_2_. Interestingly, we observed a higher NO production in EOCs with both treatments as compared to only E_2_- stimulated EOCs. In contrast, there was no further increase in eNOS phosphorylation (Fig. 6a,b). This was associated with a further decrease of active caspase 3 expression (Fig. 6c), without any effect on Bcl-2/ Bax ratio (Fig. 6d). These results suggest that there is a partially additive effect of eNOS transfection and E_2_ treatment on NO production and cell survival in EOCs.

### eNOS transfection and E_2_ stimulation of EOCs do not have an additive effect on hCASMC migration and proliferation

**Figure 7:**
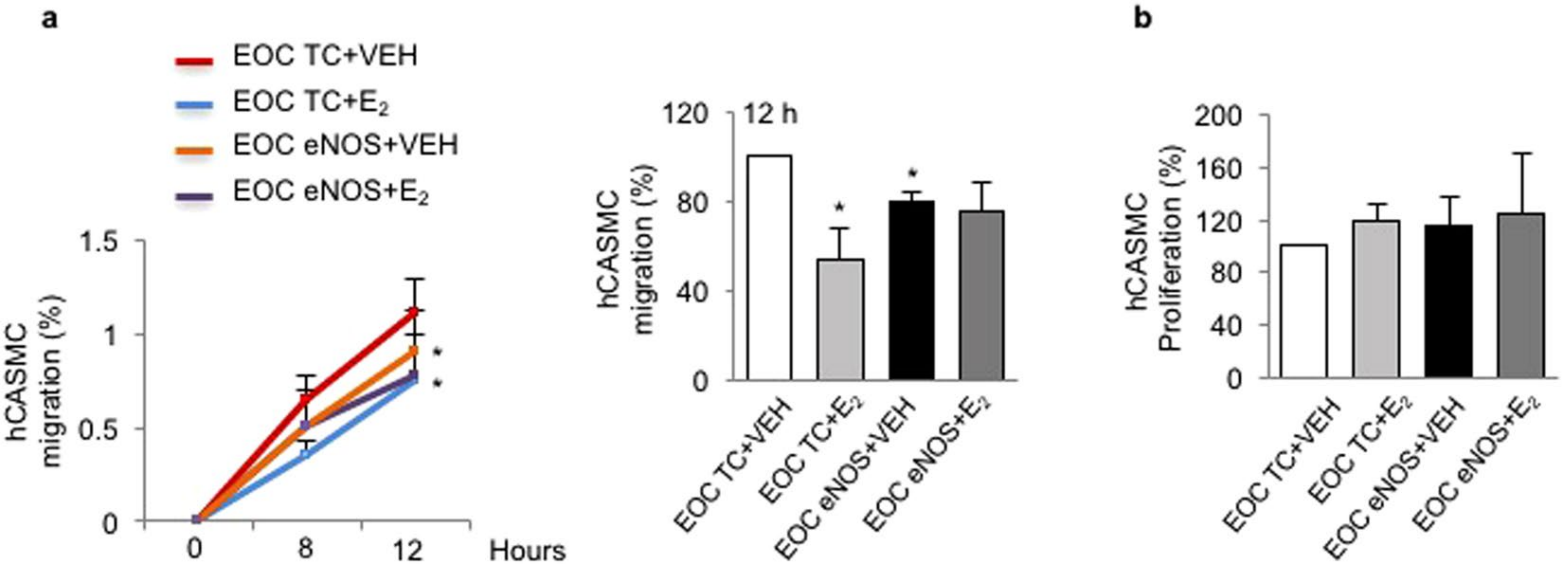
eNOS upregulation by plasmid transfection and estrogen do not have any additive effect on SMC migration and proliferation in EOCs. Human EOCs were transfected with a transfection control plasmid (EOC TC) or eNOS plasmid (EOC eNOS) or and stimulated with vehicle (VEH) or estrogen (E_2_, 10^−9^ M) for 24 hours. They were then co-cultured with human coronary artery smooth muscle cells for up to 12 hours (**a**) or 24 hours (**b**). (**a**) SMC migration was assessed by scratch assay at 8 and 12 hours. (**b**) SMC proliferation was measured by Ki67 immunostaining. Data are mean ± SEM of n = 6, *p < 0.05 vs EOC TC + VEH.

To determine whether there is an additive effect of eNOS transfection and E_2_ stimulation on hCASMC migration or proliferation, EOCs were transfected with eNOS plasmid and stimulated with E_2_ before being co-cultured with hCASMCs. We did not observe any supplemental effect of combined treatments on hCASMC migration or proliferation (Fig. 7). These results indicate that despite an increased NO production in EOCs both transfected with eNOS and treated with E_2_, there is no further effect on hCASMC function.

### Intact or eNOS-transfected EOCs do not have any effect on ERK1/2 or FAK phosphorylation in hCASMCs

To investigate the molecular mechanism involved in the inhibitory effect of eNOS-transfected or E_2_-stimulated EOCs on hCASMC function, we investigated FAK and ERK1/2 phosphorylation, two pathways involved in cell migration and proliferation. EOCs were transfected with eNOS or transfection control plasmid, or were stimulated with E_2_, or received both treatments prior to co-culture with hCASMCs. In some experiments, platelet-derived growth factor (PDGF, 10^−7^ M) was added at the time of co-culture.

A basal level of FAK phosphorylation was observed in SMCs, and no further effect of PDGF was noted (Supplemental Figs 1–3). In comparison, ERK1/2 activity was increased in SMCs stimulated with PDGF. We did not observe any effect of EOC co-culture on hCASMC FAK or ERK1/2 phosphorylation. Moreover, neither eNOS transfection nor E_2_ stimulation of EOCs, nor the two combined, had any impact on SMC FAK or ERK1/2 signaling. These results suggest that the inhibitory effect of eNOS overexpressing EOCs on hCASMC migration is not through FAK or ERK1/2 pathways.

## Discussion

This study demonstrates that human eNOS-overexpressing EOCs have increased survival rate and demonstrate improved functionality through paracrine effects. Frequently termed “endothelial progenitor cells”, circulating EOCs are defined according to surface markers and properties. One recent classification distinguishes EOCs, which display mostly paracrine actions, from late outgrowth cells, which are characterized by high proliferative potential^23^. EOCs are derived from the bone marrow and are involved in postnatal vasculogenesis and reendothelialization after endothelial damage^24,25^. EOCs have been used as a therapeutic tool in multiple clinical studies, but the therapeutic benefit of these cells is attenuated by age and disease^26,27^. Even in the absence of such mitigating factors, our data demonstrates that naïve EOCs have little influence on SMC migration or proliferation *in vitro*, but that increasing eNOS production by EOCs through transfection or estrogen stimulation significantly enhances their effects, particularly as relates to curtailing SMC migration.

NO is a well-known anti-proliferative and pro-survival factor and its effects on SMC proliferation and migration are extensively reported in the literature^28^. Therefore, the use of EOCs engineered to produce more NO was a logical step to improve effectiveness of these cells. Overexpression of eNOS or exogenous estrogen has previously been shown to enhance the capacity of bone marrow-derived EOCs to abate neointimal hyperplasia^7,29–31^. This protective effect was attributed to enhanced re-endothelialization, but no effects on SMCs were considered. Gene transfer of eNOS in bone-marrow derived mesenchymal stem cells was also found to improve cardiac repair after myocardial infarction^32^. Similarly, in models of pulmonary arterial hypertension, injection of fibroblasts^33^, mesenchymal stem cells^34^, or bone marrow-derived endothelial-like progenitor cells^35^ transfected with eNOS improved recovery, compared with null plasmid transfected-cells which allowed the disease to progress. Hence, many studies report a reparative effect of EOCs in models of vascular disease, but how these cells act on underlying SMCs has been poorly investigated.

For one, the magnified effects of eNOS-overexpressing EOCs be contingent on their increased viability. There is indeed evidence that the beneficial effects of some drugs, including estrogen, may hinge on NO-dependent reduced senescence and apoptosis of EOCs^29–31^. Depending on the dosage and the presence or not of stimulatory co-signals, NO has been shown to block or to induce apoptosis^36^. In stem cells, NO may induce auto-protection by decreasing the activity of caspase 3 through direct interaction^37^. The increase in EOC survival by eNOS/ NO pathway stimulation could be clinically very relevant since one of the critical limitations for the therapeutic application of postnatal EOCs is their low number in the circulation, which is even lower in patients with cardiovascular risk factors^38^. In this regard, we evaluated the effect of eNOS stimulation and overexpression on EOC survival. Following estrogen treatment or eNOS plasmid transfection, total and phosphorylated eNOS levels, as well as NO production, were increased in EOCs. We observed a significant decrease in cleaved caspase 3, accompanied by an enhanced Bcl-2/Bax ratio in these cells compared with controls. Combining eNOS transfection and estrogen treatment significantly increased NO production and reduced caspase 3 activity. Hence, the results suggest that in our experimental conditions, enhancing NO reduces EOC apoptosis.

Thereafter, we investigated the potential effects of native, estrogen-stimulated and eNOS over-expressing EOCs on SMC function. We first confirmed that the NO donor SNAP inhibited proliferation and migration of vascular SMCs in a dose dependent manner, in line with previous studies^39–41^. We then found that co-culture of SMCs with native EOCs did not affect SMC proliferation or migration, but it did sensitize the cells to SNAP, reducing the concentration of the NO donor required to abate SMC migration and proliferation. Although Cui *et al*. showed *in vivo* that transplantation of native EOCs reduced neointimal hyperplasia in denuded rat carotid arteries^7^, our results suggest that *in vitro*, native EOC NO production is not sufficient to regulate the activity of neighboring cells. The next step was to investigate the effect of NO-overproducing EOCs on SMC migration and proliferation. Interestingly, we observed a significant decrease in migration but not proliferation in SMC co-cultured both with estrogen-stimulated and with eNOS-transfected EOCs. In support of our results, multiple NO donors were reported to inhibit SMC migration in a concentration-dependent, reversible, and non-cytotoxic fashion^42^. However, the absence of effect on SMC proliferation suggests that higher NO levels might be required to modulate SMC proliferation *in vitro*. Indeed, Mooradian *et al*. studied the effect of different NO donors on SMC growth and showed that the inhibitory effect depended on the total amount of NO delivered, the rate of delivery, and the period of exposure to NO^43^. In our experiments, even combining estrogen treatment and eNOS overexpression failed to meet the minimum threshold required for inhibition of proliferation.

We further investigated the molecular pathway involved in the inhibitory effect observed on SMC migration by EOCs. Platelet-derived growth factor (PDGF) is a potent inducer of SMC proliferation and migration, frequently involved in neointima formation^44^. It acts by binding to its receptor that initiates a cascade of signals leading to the activation of the ERK pathway^45^. PDGF receptors have been shown to be localized to focal adhesions and stimulate focal adhesion kinase (FAK), which is linked not only to activation of ERK^46^ but also to integrin-dependent SMC migration^47^. PDGF-dependent SMC proliferation and migration is reduced by NO^48^, and recent data have revealed that even native EOCs were shown to attenuate the response of SMCs to PDGF^49^. We investigated ERK1/2 and FAK phosphorylation in SMCs co-cultured with native, estrogen-stimulated, or eNOS-transfected EOCs. Under basal conditions, EOCs did not have any effect on ERK1/2 or FAK phosphorylation, suggesting that even in the absence of PDGF the inhibitory effects of enhanced EOCs on SMC migration are unlikely to have involved FAK. Furthermore, PDGF activated ERK1/2 in SMCs, and the presence of any EOCs did not abolish this effect. Thus, not only did EOCs fail to reduce SMC proliferation in basal conditions, but even in conditions of accentuated SMC proliferation (with PDGF), EOCs are unlikely to have an effect.

Many vascular diseases are exacerbated by uncontrolled SMC proliferation and migration, including restenosis after angioplasty^50^ and pulmonary hypertension^51^. In such conditions, strategies have been devised to optimize the therapeutic potential of EOCs. Hence, it was reported that angioplasty stents coated with anti-CD34 antibodies, designed to capture circulating EOCs, induced the rapid establishment of a functional endothelial layer early in the damaged area with minimal inflammation^24^. More relevant to the current work, eNOS-overexpressing EOCs were recently found to be well tolerated in patients with pulmonary arterial hypertension, as part of a first phase trial to establish the clinical applicability of such a strategy^20^. Thus, understanding to what extent eNOS-transfected EOCs can act on SMCs is a priority.

This study comprises some limitations, however. For one, we did not uncover the mechanism whereby estrogen-stimulated or eNOS-overexpressing EOCs repress SMC migration, other than to rule out FAK and ERK signaling. This is surprising, given that FAK and integrin activation have long been associated with SMC migration^52,53^. Another potential pathway to consider is the Rho kinase signaling cascade, recently shown to be activated in SMCs in a model of pulmonary hypertension, and to be sensitive to eNOS-activating treatment^51^. Another point to consider is that SMCs contribute to atherosclerotic plaque stability^53^, and that limiting their migration may impact vulnerability. Nevertheless, this could be offset by integration of EOCs themselves into atherosclerotic lesions, participating in vascular repair^54^.

In summary, here we show for the first time that NO overproducing EOCs have a better survival and decrease SMC migration through paracrine effects. These data are potentially clinically relevant since they determine the potential of primed EOCs to prevent pathological vascular remodeling.

## Methods

All methods were carried out in accordance with relevant guidelines and regulations.

### Mononuclear cell isolation and EOC characterization

In order to isolate human EOCs, peripheral blood was obtained from healthy volunteers, (age 20–45 years, male and female, no known disease or medication). All experimental protocols including blood cell donation were approved by the research review committee of the Lady Davis Institute for Medical Research. Informed consent was obtained from all donors. EOCs derived from male and female donors produced comparable results, even in estrogen stimulation experiments. Hence, cells from both genders were pooled.

The n value is the number of times each experiment was repeated. Each individual experiment was performed with a distinct EOC population grown from cells isolated from one individual.

Twenty ml citrate-buffered blood was mixed with 15 ml of phosphate buffered saline. Then, 15 ml ficoll (Sigma-Aldrich) was carefully overlaid with the diluted blood and centrifuged for 20 minutes. The mononuclear cells (MNCs) found in the interphase were carefully collected. MNCs (5 × 10^5^) were seeded on fibronectin-coated plates in endothelial basal medium (EBM) with supplements (EGM-2, Lonza), as previously described^9^.

After 7 days of culture, EOCs were characterized as previously described^9^. Fibronectin-adherent cells were stained with 1,1′-dioctadecyl- 3,3,3′,3′ tetramethylindocarbocyanine (Dil)-labeled acetylated low-density lipoprotein (acLDL; Invitrogen) and FITC-labeled lectin from *Ulex europaeus* agglutinin (Sigma). Cells double-positive for Dil-acLDL and lectin staining were considered to be early outgrowth cells. Quantitative immunocytochemical analysis^55^ revealed that Dil-acLDL/lectin double-positive cells express the endothelial markers VEGF receptor-2 (100 ± 0%), phospho-endothelial NOS (100 ± 0%), CD31 (84 ± 8%) and to some degree the stem cell marker CD34 (18 ± 1%).

### EOC transfection

On day 4 of culture MNCs were transfected according to the following protocol: 3 μl of X-tremeGENE 9 transfection reagent (Roche) were mixed for every 100 μl of EBM (Lonza). Plasmid DNA was then added in a 3:1 ratio (μl of transfection reagent:μg of plasmid DNA) and incubated for 15 min at room temperature. The transfection complex was added in a dropwise manner on the cell culture in a 1:10 volumetric ratio (transfection complex:cell medium). Cells were transfected with either dialyzed pVAX1-heNOS plasmid DNA or pmaxFP-Green-C (Lonza) plasmid as a transfection control. Cells were cultured for 48 h after transfection.

### 17 β-estradiol stimulations

On day 6 of culture MNCs were treated with 1–100 nM E_2_ (Sigma-Aldrich) for 30 minutes (for time course experiments). To prevent hCASMC E_2-_derived stimulation in the EOC- hCASMC co-culture setting; E_2_ treated EOCs were previously washed with PBS, placed in an incubator for 30 min with 10 nM of the estrogen receptor inhibitor fulvestrant (Sigma-Aldrich) followed by another wash prior the co-culture.

### Western blotting

Cultured EOCs were harvested with a cell lysis buffer (HEPES 10 nM pH 7.4, Na pyrophosphate 50 nM, NaF 50 nM, Na_2_ EDTA 5 nM, EGTA 5 nM, Triton X-100 0.5%, Na_3_VO_4_ 2 nM, PMSF 1 nM, leupeptin 1 μg/ml, aprotinin 1 μg/ml). Protein levels were quantified by Bradford assay (Bio-Rad). The appropriate volume of SDS loading buffer (375 mM Tris-HCl pH 6.8, 6% SDS, 48% glycerol, 9% 2-mercaptoethanol, 0.03% bromophenol blue) was added to protein samples, heated for 5 min at 100 °C and loaded into 8% polyacrylamide gel. Proteins were transferred overnight to a PVDF membrane (Bio-Rad). The membrane was blocked for 1 hour at room temperature with blocking buffer (5% non-fat dry milk in TBS-T (50 mM Tris pH 7.6, 150 mM NaCl, Tween-20 0.05%)) and then incubated overnight with the appropriate primary antibodies. The membrane was washed 3 times with TBS-T and incubated with the appropriate HRP secondary antibodies (Santa Cruz goat anti-rabbit or goat anti-mouse). Next the membrane was incubated with a western lightning plus-ECL solution (Perkin Elmer). The chemiluminescence was measured using Chemidoc XRS + system (Bio-Rad) and quantified by densitometry using Quantity One software (Bio-Rad).

### BrdU assay

Primary human coronary artery smooth muscle cells (hCASMC) (PromoCell) were grown to ~70% confluency in EGM-2. After 24 h serum starving, BrdU reagent (EMD Millipore) was added at 1/3000 final concentration and cell culture inserts (BD Biosciences) containing 1 million EOCs were immediately placed over the hCASMC. After 24 hs of co-culture, EOCs were discarded; hCASMC were incubated with a fixing solution following an incubation with 1/200 BrdU detector antibody for 1 hour and then with a goat anti-mouse IgG peroxidase conjugate. Cells were then washed as described and incubated with 200 μl per well of TMB peroxidase substrate. The luminescence was measured at 450 nm with a fluorescence plate reader (BMG Labtech FLUOstar Optima).

### Ki-67 staining

hCASMC were seeded at an appropriate density on cover slips (Fisher Scientific). Cells were fixed with 2% paraformaldehyde and permeabilized with 0.5% Triton X-100 for 10 min. Cells were blocked with 3% BSA overnight at 4 °C, then rinsed with PBS and incubated with 1/50 diluted rabbit anti-Ki-67 antibody (Abcam) for 90 min followed by a fluorescent secondary antibody (Invitrogen). Images were taken using a Leica DM2000 fluorescent microscope (Leica Microsystems) and an Infinity3-1UC color camera (Leica Microsystems). Ki-67 positive cells were quantified as a percentage of total DAPI stained nuclei and normalized with either untreated EOCs or transfection control EOCs (basal conditions). For each experimental condition, an average of 200 cells were counted from 3–4 microscopic fields, and ~15% cells in each field were Ki-67+.

### Wound scratch assay

hCASMC were seeded in a 24 well plate in EGM-2 medium. After a 24-hour starving cells were wounded once with a small tip by scratching across the maximum diameter of each well. Pictures were taken immediately after scratching (time 0). Cell culture inserts containing EOCs were placed over the hCASMC. 30 minutes later, 0.1 μM PDGF (R&D Systems) or the nitric oxide donor, S-nitroso-N-acetylpenicillamine (SNAP, Sigma Aldrich) was added as needed. pictures were also taken at 8 and 12 hours with EOC co-culture. Images were analyzed using Image J software by measuring the size of the denuded area.

### Flow cytometry

Cells were incubated with human FcR blocking reagent (Miltenyi Biotec), then fixed and permeabilized (BD Cytofix/Cytoperm Fixation/Permeabilization). Cleaved caspase-3 antibody (Cell Signaling) was added at a concentration of 1/50. Cleaved caspase-3 positive cells were quantified using a FACSCalibur flow cytometer. Results were analyzed using CellQuest Pro software (BD Biosciences).

### NO probe

MNCs were seeded on fibronectin coated cover slips. On day 6, DAF-FM diacetate (Molecular Probes) was added for 1 hour at 37 °C. Then, cells were fixed with 2% paraformaldehyde. Cover slips were mounted on slides using a mounting medium containing DAPI. Images were taken using an Infinity 3 Leica fluorescent microscope. NO-positive cells were quantified as ratios of DAF-DM/Dapi positive cells and normalized with untreated EOCs (for E2 time course experiments) and expressed as percentage of EOCTC (for eNOS overexpressing experiments). A single threshold level was applied to all experiments and all conditions for analysis of DAF-FM levels (ImageJ). For each experimental condition, an average of 265 cells were counted from 4 microscopic fields.

### Statistical Analysis

All data are presented as mean ± standard error of the mean (SEM). We performed a Student’s t-test to compare two groups, or a two-way ANOVA followed by the Newman-Keuls post-test for multiple group comparisons. Probability values of p ≤ 0.05 were considered to be statistically significant.

## Electronic supplementary material

Supplementary figures
