# Supplementary material for: Endothelial nitric oxide synthase overexpressing human early outgrowth cells inhibit coronary artery smooth muscle cell migration through paracrine functions
Source: Sci Rep. 2018 Jan 17;8:877. doi: 10.1038/s41598-017-18848-z (PMC5772515; doi:10.1038/s41598-017-18848-z)
Supplement: Supplementary file 1 — Supplementary figures [file 41598_2017_18848_MOESM1_ESM.pdf]

Supplementary figures

Endothelial nitric oxide synthase overexpressing human early outgrowth cells inhibit coronary artery smooth muscle cell migration through paracrine functions

Sergio Guber[1], Talin Ebrahimian[1], Maryam Heidari[1], Nicoletta Eliopoulos[1], Stephanie Lehoux[1,*]
[1]Lady Davis Institute for Medical Research, McGill University, Montreal, Quebec, Canada.

***Corresponding author:**

Stephanie Lehoux
stephanie.lehoux@mcgill.ca
Lady Davis Institute for Medical Research
3755 Cote Ste Catherine, Montreal, QC, H3T 1E2, Canada
Phone: +1-514-340-8222; fax: +1-514-340-8252

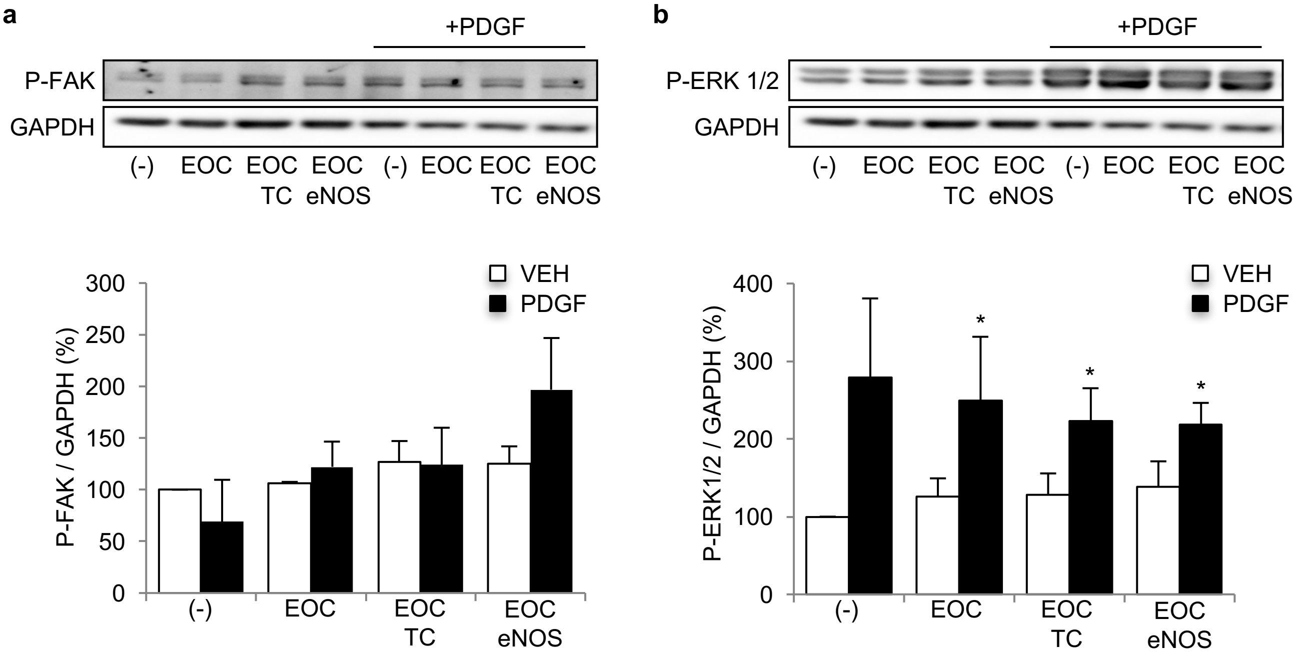

Supplemental Fig 1. Intact or eNOS transfected EOCs do not have any effect on FAK or ERK1/2 phosphorylation in SMCs.

Human EOCs were left intact (EOC), or transfected with a transfection control plasmid (EOC TC) or eNOS plasmid (EOC eNOS). Then they were then placed in co-culture with SMCs and cells were stimulated with vehicle or PDGF (10^{-7}M) for 10 minutes. Phosphorylated (P) FAK (Y395) **(a)** and phosphorylated ERK1/2 **(b)** expression were evaluated by western blot. Data are mean ± SEM of n=6, *p<0.05 vs no EOC (-).

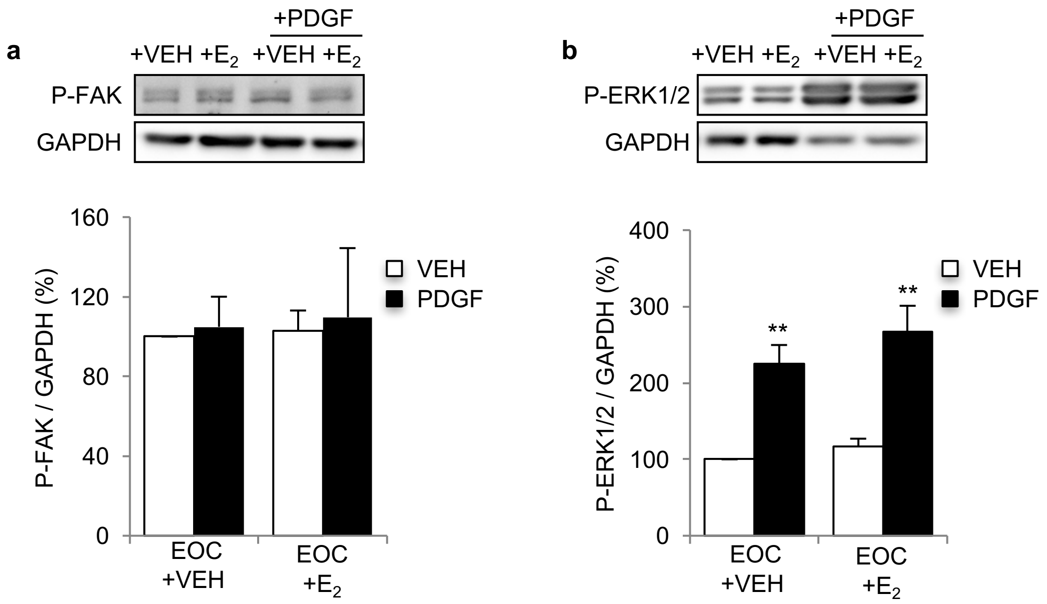

Supplemental Fig 2. Estrogen pre-stimulated EOCs do not have any effect on FAK or ERK1/2 phosphorylation in SMCs.

Human EOCs were pre-stimulated with vehicle (VEH, DMSO) or estrogen (E_2, 10^{-9}M) for 24h. They were then placed in co-cultured with SMCs and cells were stimulated with vehicle or PDGF (10^{-7}M) for 10 minutes. Phosphorylated (P) FAK (Y395) **(a)** and phosphorylated ERK1/2 **(b)** expression were evaluated by western blot. Data are mean± SEM of n=6, **p<0.01 vs EOC +VEH.

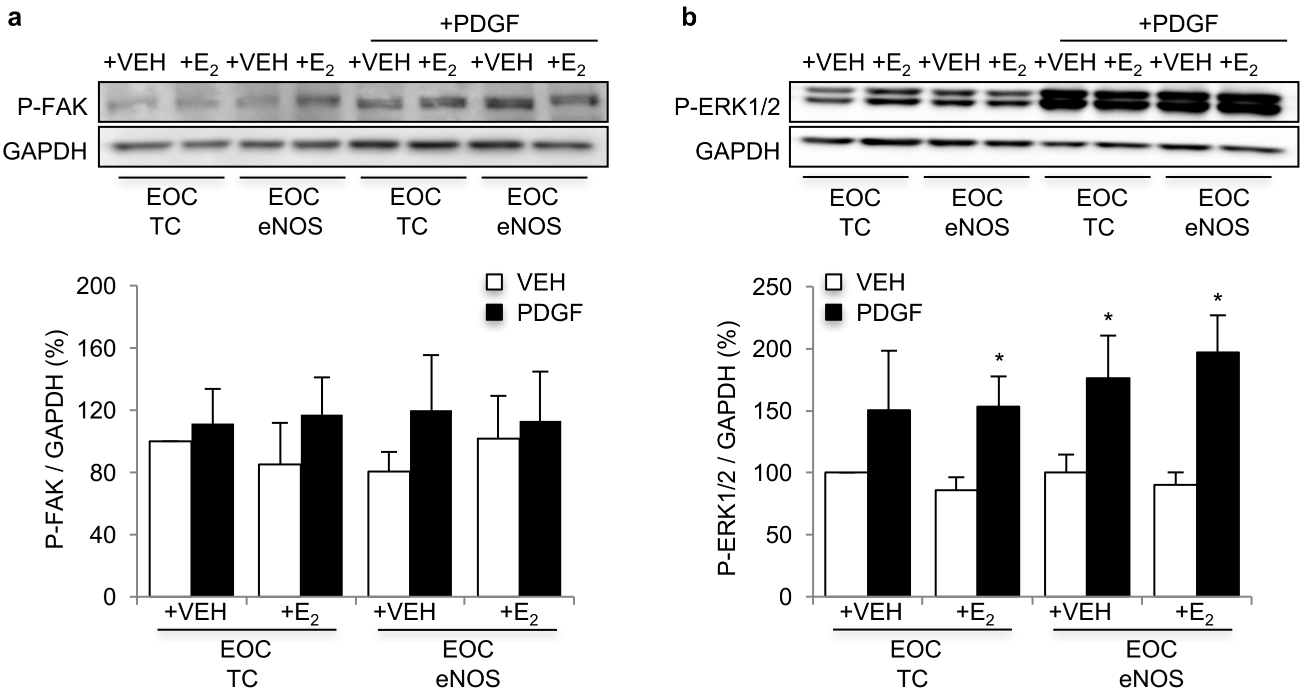

Supplemental Fig 3. eNOS-transfected and estrogen-stimulated EOCs do not have any effect on FAK or ERK1/2 phosphorylation in SMCs.

Human EOCs were transfected with a transfection control plasmid (EOC TC) or eNOS plasmid (EOC eNOS) and stimulated with vehicle (VEH, DMSO) or estrogen (E_2, 10^{-9}M) for 24h. They were then placed in co-culture with SMCs and cells were stimulated with vehicle or PDGF (0.1µM) for 10 minutes. Phosphorylated (P) FAK (Y395) **(a)** and phosphorylated ERK1/2 **(b)** expression were evaluated by western blot. Data are mean ± SEM of n=6, *p<0.05 vs EOC +VEH.

a

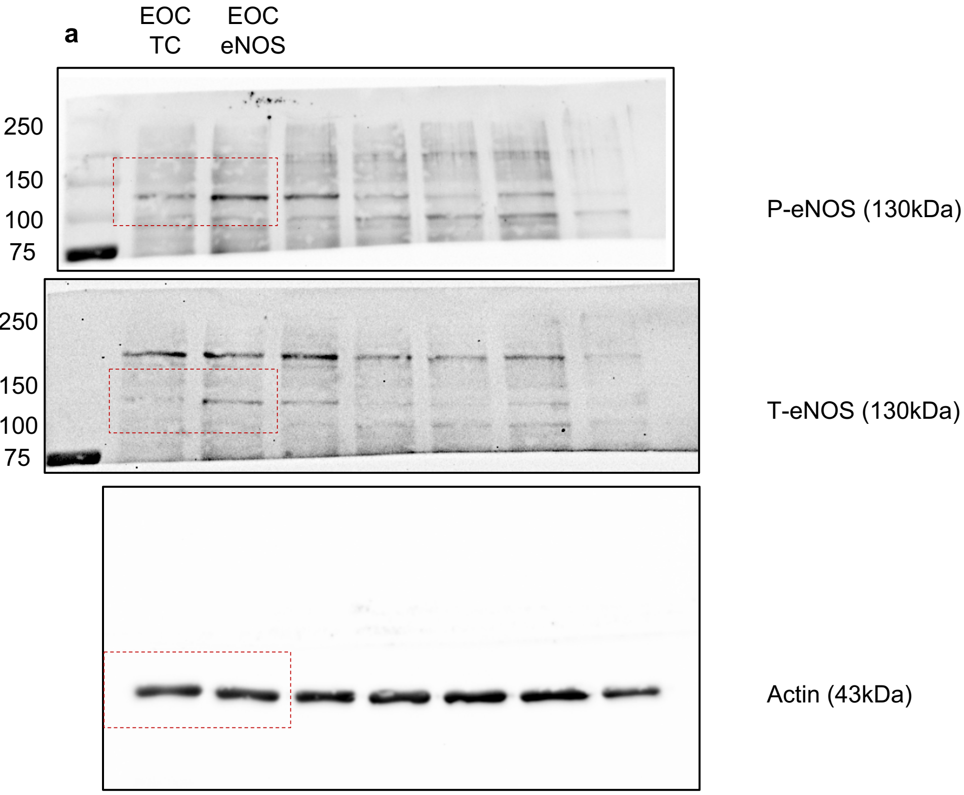

b

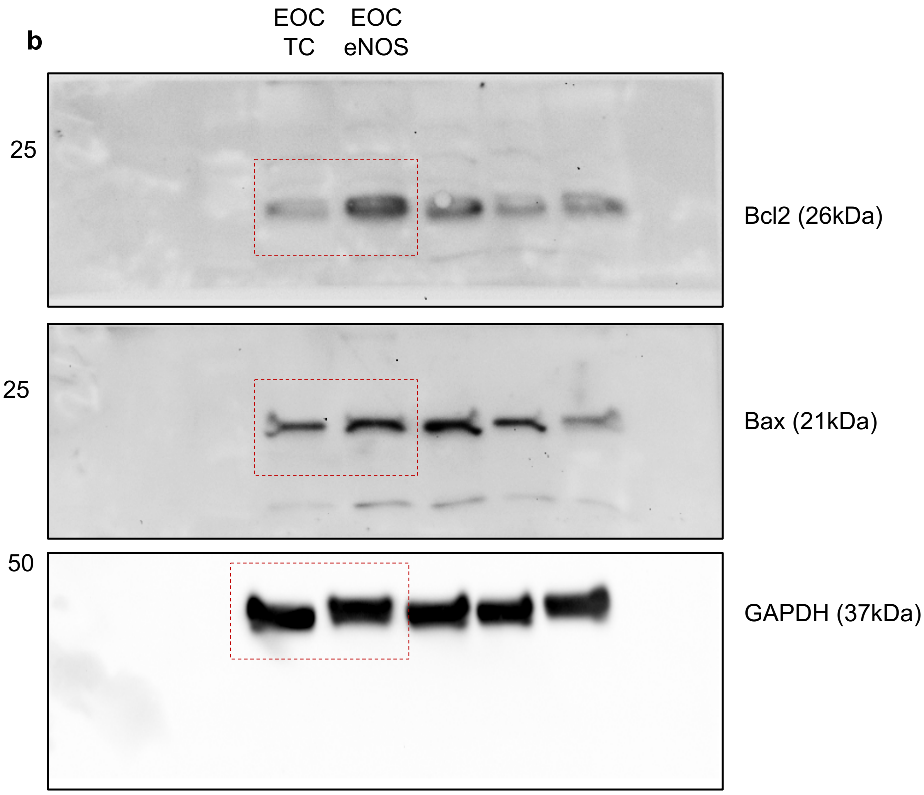

Supplemental Fig 4. Uncropped western blot scans of Figure 2
a: Representative images of western blots for Total (T) and phosphorylated (pS1177) (P) eNOS (a) Bcl-2, Bax (b) and actin//GAPDH. The red squares represent cropped images represented in figure 2 a and d.

a

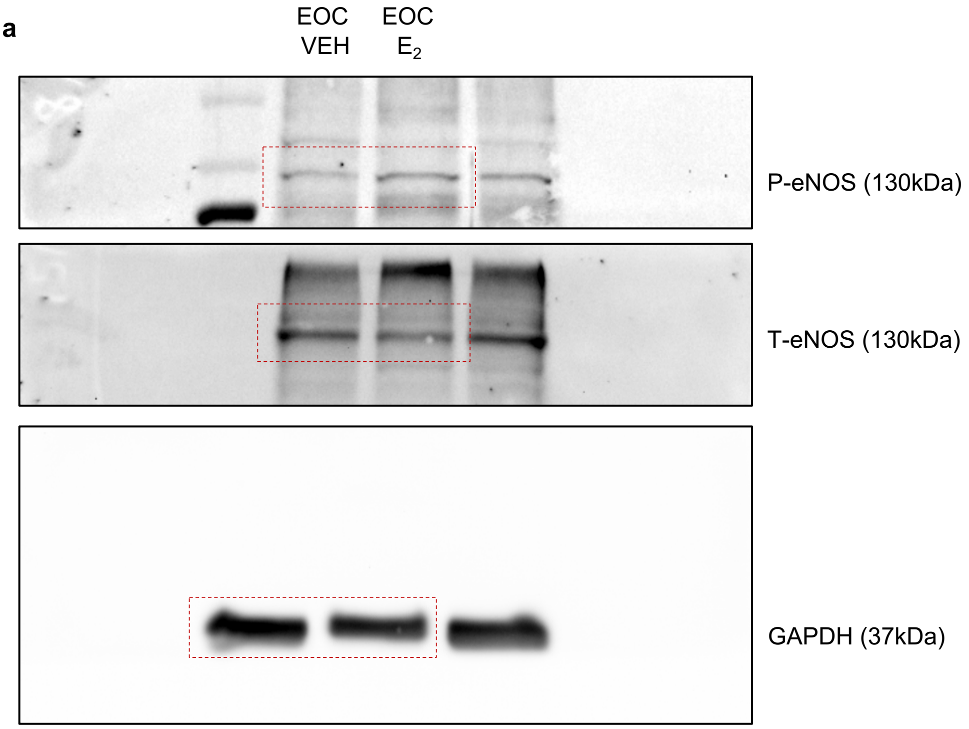

b

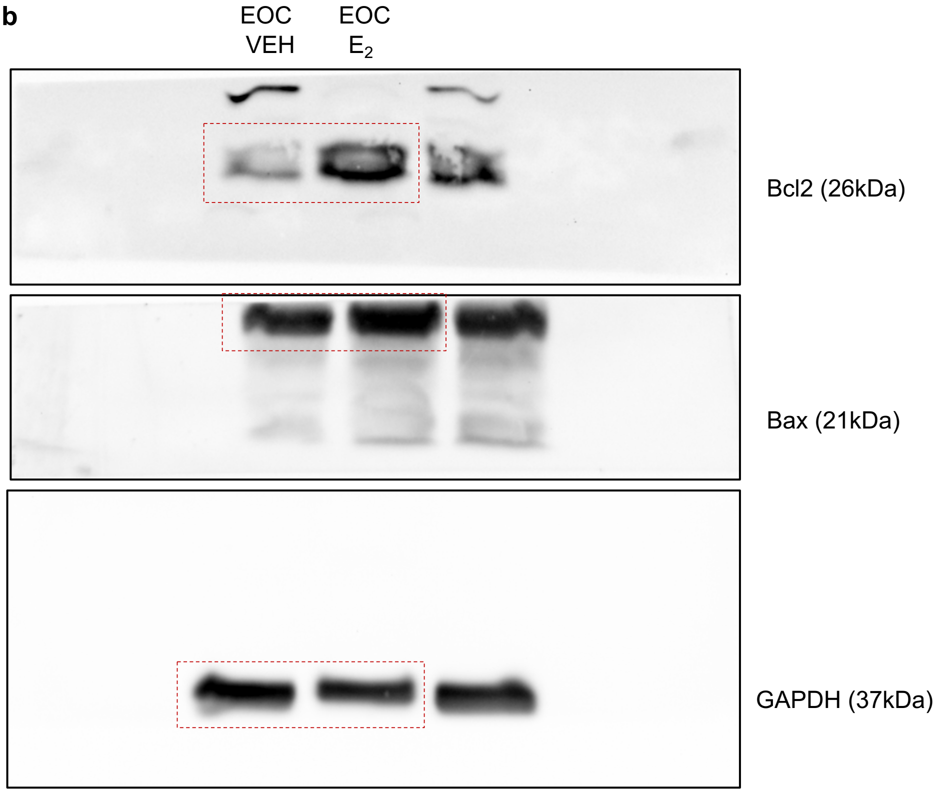

Supplemental Fig 5. Uncropped western blot scans of Figure 4

a: Representative images of western blots for Total (T) and phosphorylated (pS1177) (P) eNOS (a) Bcl-2, Bax (b) and GAPDH. The red squares represent cropped images represented in figure 4 b and c.

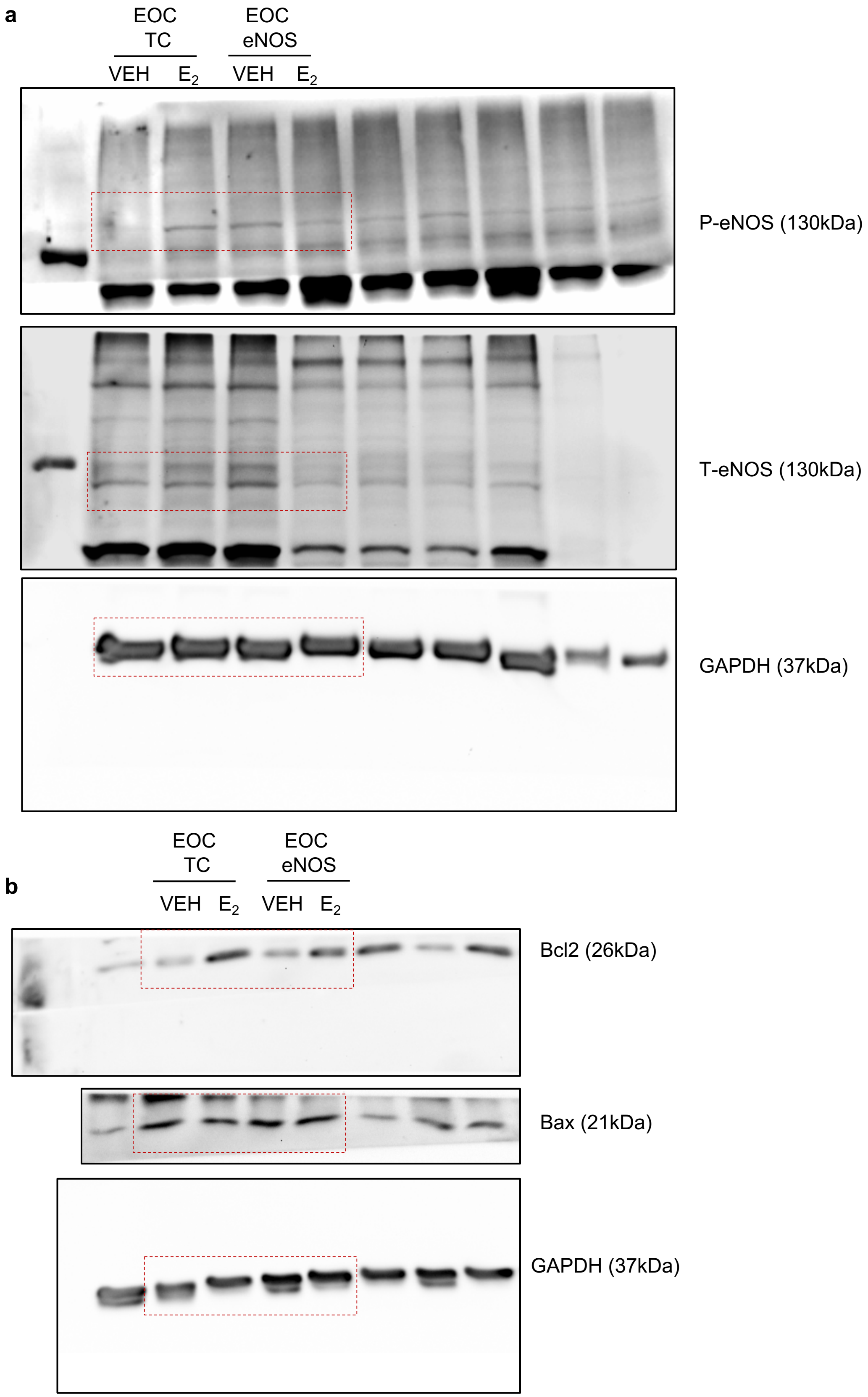

Supplemental Fig 6. Uncropped western blot scans of Figure 6

a: Representative images of western blots for Total (T) and phosphorylated (pS1177) (P) eNOS (a) Bcl-2 and Bax (b) and GAPDH. The red squares represent cropped images represented in figure 6 a and d.

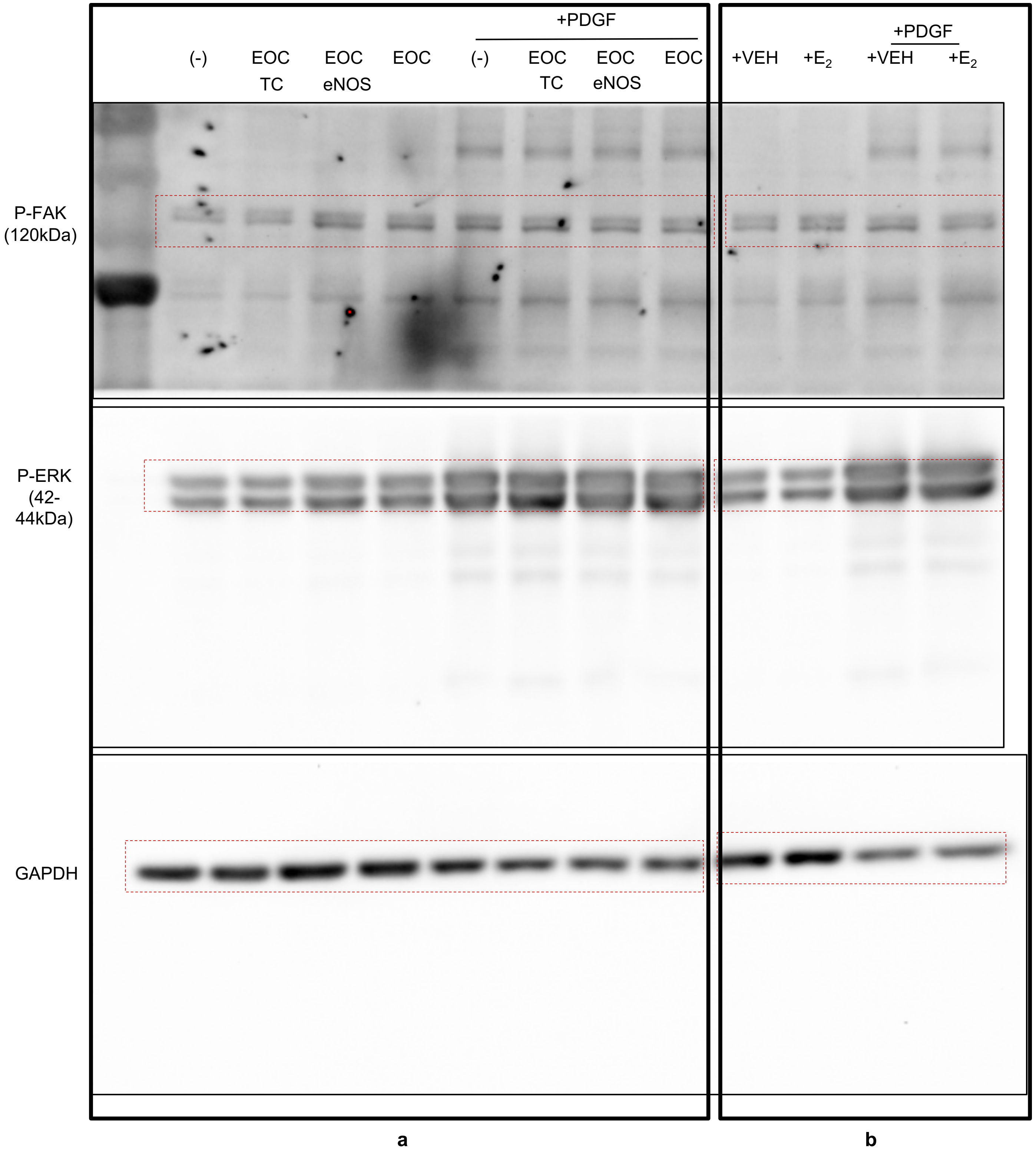

Supplemental Fig 7. Uncropped western blot scans of supplemental figure 1,2
Representative images of western blots for Phospho-FAK (Y395), –ERK1,2 and GAPDH. **a:** Images represented in supplemental figure1. **b:** Images represented in supplemental figure 2.The red squares represent cropped images represented in supplemental figure 1 and 2.

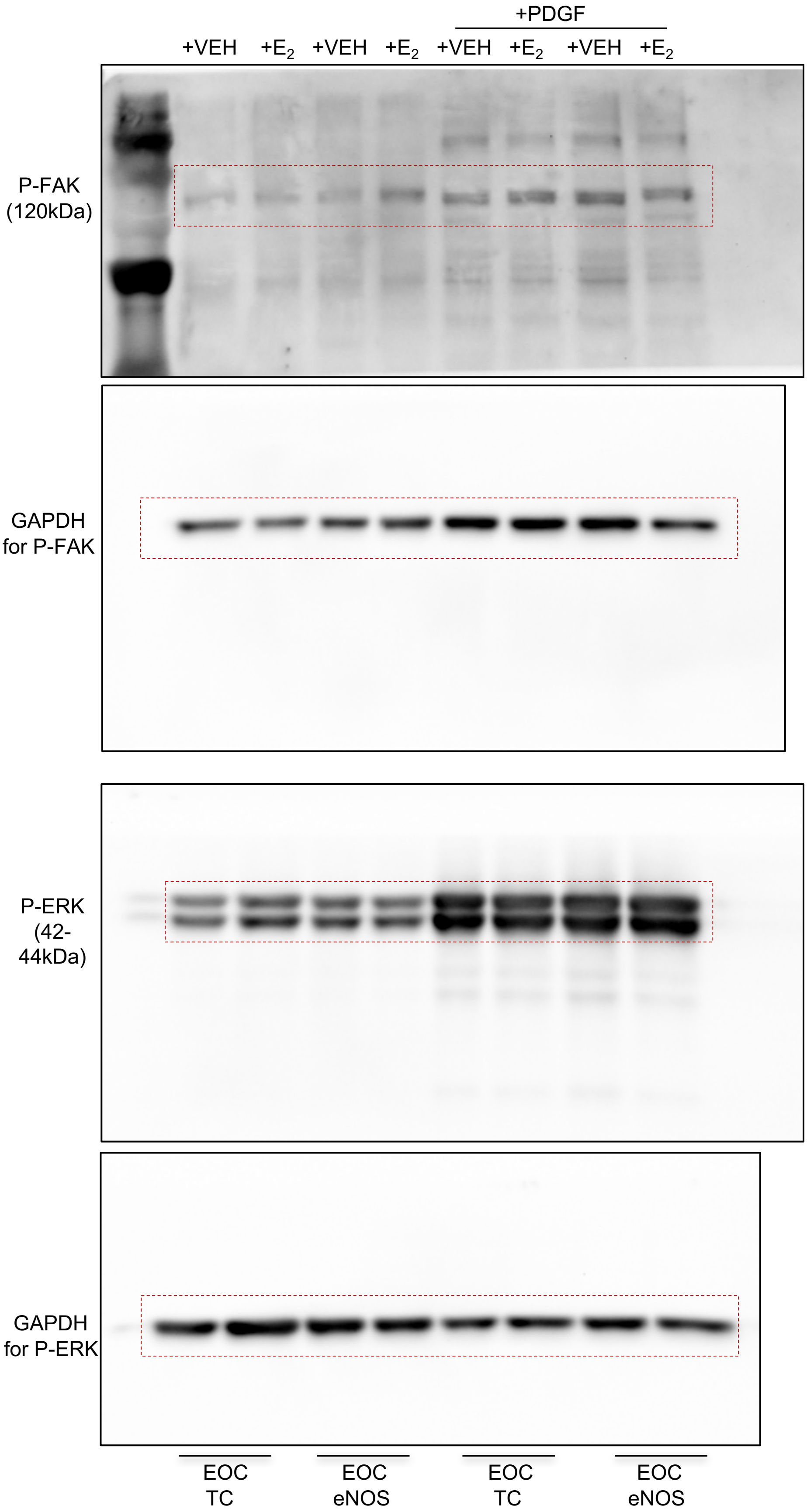

Supplemental Fig 8. Uncropped western blot scans of supplemental figure 3.
Representative images of western blots for Phospho-FAK (Y395), –ERK and GAPDH. The red squares represent cropped images represented in supplemental figure 3.
